# Drivers of the Intention to Receive a COVID-19 Booster Vaccine: Insights from the UK and Australia

**DOI:** 10.3390/vaccines10101730

**Published:** 2022-10-17

**Authors:** Kirsten Barnes, Ben Colagiuri

**Affiliations:** School of Psychology, University of Sydney, Sydney, NSW 2006, Australia

**Keywords:** vaccination, SARS-CoV-2, COVID-19, vaccine hesitancy, vaccine intention, booster vaccine, psychological predictors

## Abstract

As the global pandemic perpetuates, keeping the population vaccinated will be imperative to maintain societal protection from the SARS-CoV-2 (COVID-19) virus. However, while empirical evidence regarding predictors of the intention to receive a first COVID-19 vaccine has amassed, our understanding regarding the psychological and behavioral drivers of continued COVID-19 vaccination remains limited. In this pre-registered study (UK: AsPredicted#78370|Australia: AsPredicted#81667), factors predicting the intention to receive a COVID-19 booster vaccine were investigated in two adult samples from the UK (*N* = 1222) and Australia (*N* = 1197) that were nationally representative on factors of age, gender, and geographic location. High levels of booster intent were found (73% and 67%, respectively). Exploratory Structural Equation Modelling (ESEM) revealed three key predictors of the intention to receive a booster vaccine that emerged across both UK and Australian samples: concern regarding the COVID-19 virus, positive perceptions of the COVID-19 vaccines, and the perceived severity of side effects experienced to the last COVID-19 vaccine dose. Several additional factors (age, months since the last COVID-19 vaccine, familiarity with side effects, and regularly receiving the influenza vaccine) were present in the Australian dataset. These findings provide important evidence that targeting psychological perceptions of the COVID-19 vaccine and virus may serve to maintain participation in the COVID-19 vaccination programme, paving the way for future behavioural research in this area.

## 1. Introduction

At the time of writing, the SARS-CoV-2 (COVID-19) virus has been circulating in the population for over two and a half years and has caused in excess of six million deaths worldwide. The future trajectory of the virus remains unclear, with some hope that it may ultimately reduce to a milder endemic state [1]. However, given that it is currently unclear how permanent immunity can be accomplished [2,3], continued monitoring of infection [4], combined with ongoing vaccine administration [5] and development [6] is likely to be necessary.

Current evidence demonstrates that the efficacy of COVID-19 vaccines wane over time [7], with booster doses necessary to increase protection [8]. However, vaccine availability does not necessarily translate to vaccine acceptance, with vaccine hesitancy causing a significant barrier to societal protection [9,10,11]. Models regarding the drivers of vaccination behaviour have suggested several pathways to vaccination, including psychological and cognitive perceptions (perceived risk of disease and vaccine confidence), social processes (social norms, health worker recommendation, and vaccine equity) and practical considerations (including access, availability, and cost) [12]. In the present study, we focus on the first of these: psychological and cognitive perceptions in conjunction with contextual factors relevant to vaccine readministration, as outlined below.

Over the past few years, considerable empirical evidence has amassed regarding the factors that predict the intention to receive the primary course of a COVD-19 vaccine. Meta-analyses of these data indicate that, beyond basic sociodemographic variables, psychological perceptions comprising concern regarding COVID-19 virus and positive perceptions of the COVID-19 vaccine, as well as situational and contextual factors such as previous COVID-19 infection, and regular experience with the influenza vaccine, are some of the strongest predictors of COVID-19 vaccine acceptance [13,14,15,16]. However, in all cases, respondents had no prior experience with a COVID-19 vaccine. In contrast, we know relatively little about the predictors of intent to receive a booster vaccine. This is important because a number of additional situational and contextual factors regarding the vaccine should feedback to inform predictors of vaccine behaviour in cases where the same vaccine is repeatedly administered [12]. These may include factors such as the experience of adverse events to the primary course of the vaccine, number of months elapsing since last receiving a vaccine, perceived need for continued vaccination due to being immunocompromised, and familiarity with information regarding potential side effects accumulating as the vaccine programme has matured. At present, however, very few studies have investigated the intention to receive a booster vaccine. A recent meta-analysis reported on just twelve studies that have investigated the predictors of booster intent, with the majority focusing on sociodemographic factors and limited to Asian samples [17]. Additionally, despite concern regarding the COVID-19 virus and positive perceptions of the COVID-19 vaccine (i.e., psychological perceptions) being some of the strongest predictors of the intention to receive a first COVID-19 vaccine, only five studies included predictors investigating the former, and four the latter. With respect to experiential factors that may feedback to modulate the intention to receive a booster vaccine, only four investigated previous adverse reactions, six prior COVID-19 infection, and three experience with the influenza vaccine. As such our understanding of the psychological and contextual factors that serve to maintain continued COVID-19 vaccination is currently lacking.

In the present study, we investigated the psychological and contextual predictors of the intention to receive a COVID-19 booster vaccine in two nationally representative samples that are currently underrepresented in terms of evidence; the United Kingdom (UK; one study in previous meta-analysis) and Australia (no studies in previous meta-analysis). As such, we focused on identifying the factors that keep previously vaccinated individuals within a given booster vaccine programme. Given that these factors are psychological in nature, these results highlight potential routes through which behavioural intervention may increase vaccination intention in future.

## 2. Materials and Methods

Ethical approval was obtained from the University of Sydney Human Research Ethics Committee (reference 2021/792 and 2021/871; UK and Australian samples, respectively) with the research pre-registered (aspredicted#78370 and #81667).

### 2.1. Participants and Design

All participants were recruited via Pureprofile, an ISO-certified panel provider, with participants screened for age, gender, and geographic location. Quotas were applied during recruitment to ensure that the samples closely matched national statistics regarding these variables. Data from the UK sample (*N* = 1222) was collected between 27 October–8 November 2021 and the Australian sample (*N* = 1204) between 3–13 December 2021. Inclusion criteria for both studies were: (1) 18+ years of age; (2) currently residing in the target country (UK|Australia); (3) self-reported English fluency; (4) two doses of the primary COVID-19 vaccines on offer at the time of testing (i.e., the Pfizer or AstraZeneca vaccine); (5) no COVID-19 booster vaccine. Please note, a minority of respondents in the Australian sample (*N* = 7; 0.6%) reported receiving both the Pfizer and AstraZeneca vaccine and were therefore removed from the sample to ensure equivalence with the UK dataset (final *N* = 1197). All participants were paid a nominal fee for their participation (£3.50/$2). It was ensured that participants had no known medical reason (e.g., allergy) that prohibited them from receiving a booster vaccine.

### 2.2. Procedure

Participants opted into the study via adverts placed on an online portal hosted by the panel provider. All participants were provided with an information statement outlining the purpose of the research and provided informed consent. Both datasets were collected as part of larger pre-registered studies (UK: AsPredicted#78369|Australia: AsPredicted#81666) investigating the effect of an intervention (i.e., side effect framing) on booster intentions (for published articles, see [18,19]). However, all variables analysed in the current observational studies were presented prior to randomisation to the intervention in these larger studies and as such are not confounded by it. Cross-sectional data were collected online via Qualtrics (UK sample) or Pureprofile’s inhouse platform (Australian sample), with the survey accessible to personal computer, tablet, and smartphone. Participants completed the survey in one sitting and could not return to the study URL. All completing participants were presented with an electronic debrief outlining the purpose of the study for download.

### 2.3. Survey

#### 2.3.1. Primary Outcome: Booster Vaccine Intention

The primary outcome in both studies was the general intention to receive a COVID-19 booster vaccine (i.e., independent of any specific vaccine type). In the UK survey this was measured via a 100-point visual analogue scale (VAS) ranging from low (*definitely won’t receive a vaccine*) to high intention (*definitely will receive a vaccine*). In the Australian survey, the same primary outcome was measured via a 5-point Likert-type scale (*definitely won’t, probably won’t, may or may not, probably will, definitely will*).

#### 2.3.2. Predictor Variables: Both Surveys

##### COVID-19 Virus and Vaccine Perceptions

Five items were adapted from the thinking and feeling category of the behavioural and social drivers (BeSD) of vaccine uptake guidelines published by the WHO [20]. Specifically, these items were chosen to represent psychological perceptions (cognitive and emotional) regarding the SARS-CoV-2 (COVID-19) virus and vaccine. Two variables concerned the perceived risk of experiencing COVID-19, either personally or amongst close friends and family: “*How concerned are you about [your close family and friends] getting COVID-19?*”. Three variables measured perceptions of vaccine trust and confidence. These concerned general vaccine trust (*“How much do you trust the COVID-19 vaccines?”*), personal vaccine confidence (*“How important do you think getting a COVID-19 vaccine will be for your health? Would you say…”*) and confidence regarding others (*“How much do you think getting a COVID-19 vaccine for yourself will protect other people in your community from COVID-19?”*). Responses were collected via 100-point VAS for consistency with the other measures in the survey. Items were selected when the guidelines were first published and therefore limited psychometric data existed. Based on theory [12], two separate latent variables were anticipated—“concern regarding the virus” vs. “perceptions of the vaccine”—and it was expected that both would be positively associated with booster intention.

##### Months since Last COVID-19 Vaccine

Booster intention was expected to increase as vaccine efficacy waned. As such, data were collected regarding the number of months elapsing since the respondents last COVID-19 vaccine (i.e., the second dose of the primary vaccine course).

##### Previous Vaccine Side Effects

The average severity of the side effects experienced after the first and second dose of the primary vaccine course were recorded as separate variables on a 100-point VAS (anchors: *‘mild’*, *‘moderate’*, *‘severe’*). The following descriptions accompanied the item to guide responses, as have been implemented elsewhere [21,22]: ‘mild’ (caused you mild distress or discomfort, but no impairment in daily functioning); ‘moderate’ (caused moderate distress or discomfort or at least some impairment in daily functioning); ‘severe’ (caused you severe distress and discomfort, severe impairment in daily functioning, or acute danger to health). Experience of adverse events was expected to reduce intention.

##### General Familiarity with COVID-19 Vaccine Side Effects

Ratings regarding perceived familiarity with the side effects associated with the AstraZeneca, Pfizer, and Moderna vaccines were collected on a 100-point VAS for each vaccine type and then averaged. These three vaccine types were selected as they formed the primary booster vaccines on offer in the UK and Australia. Item wording was as follows: *“Please rate your familiarity with the side effects of the following COVID-19 vaccines*”. Given information circulating regarding severe side effects, it was anticipated that familiarity with these side effects would decrease booster intention.

##### Previous Vaccine Type

Whether respondents reported receiving the AstraZeneca or Pfizer vaccine as their primary course of a COVID-19 vaccine was recorded to determine whether any differences in predictors existed dependent of vaccine history.

##### Age

Given that significantly greater health risks associated with infection from the SARS-CoV-2 (COVID-19) virus exist among older adults, age (measured in years at the time of data collection) was employed to predict booster intention, with increased intention expected with age. However, the focus of the present study was on psychological and situational predictors, rather than sociodemographic ones.

#### 2.3.3. Predictor Variables: UK Survey

##### Experience with the COVID-19 Virus

Items were employed to capture personal exposure to COVID-19, as well as exposure through close friends and family. Item wording (*To your knowledge, are you, or have you been, infected with COVID-19?/To your knowledge, have any of your close family members or friends been infected with COVID-19?*) was taken from the WHO ‘Behavioural and Social Drivers of Vaccination Guidebook’ [20]. Because of the low number of COVID-19 cases in Australia at the time of data collection, these variables were pre-registered only for descriptive purposes in this survey (see [19]) and not as a predictors. As personal experience with the COVID-19 virus has been found to be positively associated with an increased intention to receive a primary course of a COVID-19 vaccine [23], a positive association between this variable and booster intention was expected (although we note that personal experience may also afford immunity, reducing motivation). A positive association was similarly predicted between witnessing close others with the virus and the intention to be vaccinated.

#### 2.3.4. Predictor Variables: Australian Survey

Two additional variables were added to the Australian survey, as outlined below.

##### Experience with the Influenza Vaccine

Experience with the influenza vaccine was measured (“*Before the COVID-19 pandemic, how regularly did you get a seasonal influenza (flu) vaccination?*”) with a top-box approach taken to compare those who received the flu vaccine ‘*every year*’ with all other responses (‘*never*’, ‘*rarely*’, ‘*usually*’). Regular experience of the flu vaccine was expected to be positively associated with booster intention.

##### Status as Immunocompromised

Booster intention among those immunocompromised was expected to be increased, given the greater risk of infection from the COVID-19 virus. Wording of the item was as follows: “*Are you regarded as immunocompromised by your GP (i.e., you have a weakened immune system due to a medical condition or treatment)*” and rated as Yes/No.

### 2.4. Statistical Analysis and Sample Size

Based on theory [12], two factors were expected to emerge from BeSD items: psychological perceptions regarding the COVID-19 virus and COVID-19 vaccine. Exploratory structural equation modelling (ESEM) [24], including observed and latent variables, was used to analyse the predictors of COVID-19 Booster Vaccine Intention (i.e., the primary outcome). However, prior to running the full models, ESEM was first employed to explore only the structural component of the model to confirm that a two-factor solution regarding the BeSD items was a better fit for the data than a single factor. ESEM was chosen as the analysis type as it allows a more flexible approach to model building, including cross-loadings of conceptually similar factors (such as those measured by the BeSD items), than traditional SEM which uses CFA to estimate latent variables [25]. An oblique target rotation method was employed, with cross-loadings between items and non-target factors constrained to be as close to zero as possible [26]. The five-point Likert-scale was used as the outcome in the Australian dataset, rather than a top-box approach as pre-registered, due to non-convergence issues associated with the latter. Maximum likelihood with robust standard errors (MLR) was selected as the estimator due to the non-Gaussian nature of the data and fully standardised parameter estimates are reported unless stated otherwise. Analysis was performed with MPlus (v.7). Model fit was assessed using the following rules of thumb for each of the following indicators: root mean square error of approximation (RMSEA) ≤0.06–0.08 [27,28], comparative fit index (CFI) ≥0.90–0.95, Tucker–Lewis index (TLI) ≥0.90–0.95 [29,30], and the Standardized Root Mean Square Residual (SRMS) of <0.08 [29]. Model building was exploratory, with the intention of achieving the most parsimonious model. As per a pre-registered stats plan, models were pruned to remove non-predictive variables, starting with the variable with the smallest standardised beta. Improvement in model fit was assessed at each iteration using the Satorra-Bentler method [31]. As there was limited evidence regarding the effect size of the variables tested on booster intent at the time of data collection, sample size was calculated based on an a priori power analysis for a small effect *f^2^* = 0.02 (95% power, alpha = 0.05) for a model that included all predictors.

## 3. Results

### 3.1. Descriptive Statistics

Table 1 presents descriptive statistics for the predictor variables included in the UK and Australian models, as well as basic demographic information (age, gender, and employment status) regarding both samples. Further information regarding sample demographics, including regional location, level of education, and self-report ethnicity, are detailed elsewhere (see: [18,19]).

### 3.2. Vaccine Intention: UK and Australia

**Figure 1:**
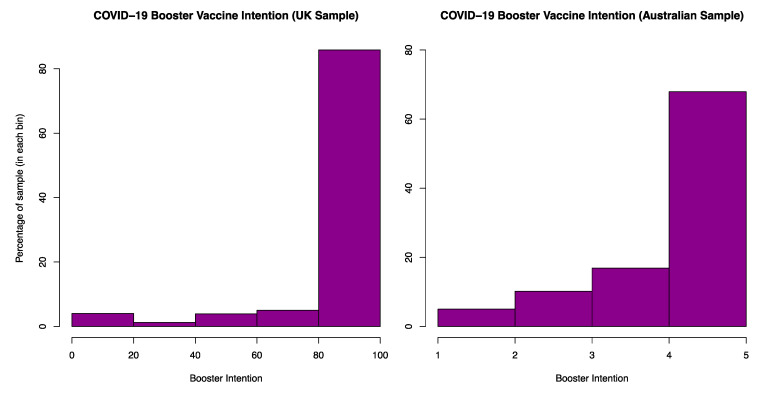
Distribution of the primary outcome (Booster Vaccine Intention) across the UK (**left**) and Australian (**right**) samples presented as the percentage of the sample in each bin of data.

Figure 1 presents vaccine intention in the UK and Australia. Intention was high in both countries (UK: *M* = 91.4, *SD* = 21.5, range = 0–100 | Australia: *M* = 4.5, *SD* = 0.9, range = 1–5) In the UK, 73% of participants reported that they ‘definitely would’ receive a booster vaccine, while 67% of respondents in the Australian sample chose this same option (although it is noted that the granularity of the two scales differed).

### 3.3. Latent Variables: Psychological Perceptions of the COVID-19 Virus and Vaccine

Across both the UK and Australian datasets, ESEM performed on the BeSD items demonstrated better fit when the model included two factors rather than one. The first factor comprised the items regarding concern about the virus and the second factor the items regarding perceptions of the vaccine. In both samples, cross-loadings for the two-factor model were found to be small (<0.12) and have residual variances >0.10 and <0.90 [25]. Model fit regarding the one and two-factor ESEM models are presented in Table 2.

### 3.4. Primary Analysis

#### 3.4.1. ESEM UK Sample

Table 3 presents the variables pruned from the UK model at each stage of refinement in order to achieve the most parsimonious model.

**Figure 2:**
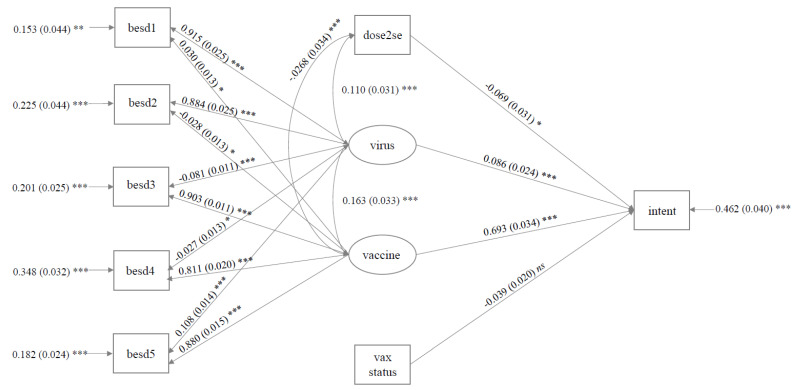
Variables in the final model concerning the UK Sample (statistics are reported as: standardised estimate (standard error) *** (*p* < 0.001) ** (*p* < 0.01); * (*p* < 0.05)). Covariance between latent variables and dose 2 side effects were included to improve fit. The latent variable ‘virus’ relates to psychological perceptions of the COVID-19 virus, and ‘vaccine’ to the COVID-19 vaccine. Other abbreviations are outlined in Table 1 above.

The final model (Stage 6) was a good fit for the data (CFI = 0.97; TLI = 0.94; SRMR = 0.04; RMSEA = 0.08) and accounted for 54% of the variance in Booster Intention (*R^2^* = 0.54, *SE* = 0.04, *p* < 0.001). As presented in Figure 2, positive perceptions regarding the COVID-19 vaccine (*β* = 0.69, *SE* = *0*.03, *p* < 0.001, 95% CI [0.63, 0.76]) and concern regarding the COVID-19 virus (*β* = 0.09, *SE* = 0.02, *p* < 0.001, 95% CI [0.04, 0.13]) positively predicted Booster Intention. Side effect severity to the second dose of the primary course (but not the first; see Model Stage 5, Table 3) negatively predicted increased Intention (*β* = −0.07, *SE* = 0.03, *p* = 0.026, 95% CI [−0.13, −0.01]). Vaccine status (AstraZeneca vs. Pfizer) neared significance, with those receiving the AstraZeneca vaccine reporting numerically higher Intention (*β* = −0.04, *SE* = 0.02, *p* = 0.056, 95% CI [−0.08, 0.001]).

#### 3.4.2. ESEM Australian Sample

Table 4 presents the variables pruned from the Australian model at each stage of refinement.

**Figure 3:**
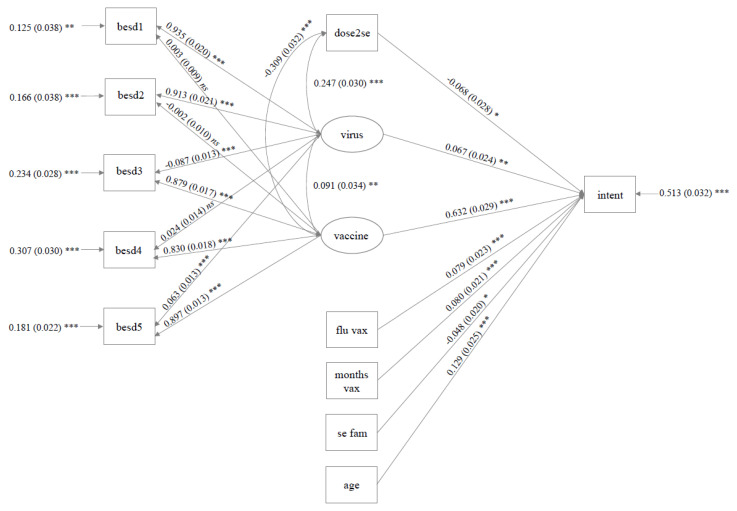
Variables in the final model concerning the Australian Sample. Note, statistics are reported as: standardised estimates (standard error) *** (*p* < 0.001) ** (*p* < 0.01); * (*p* < 0.05). As in the UK model, covariance between latent variables and dose 2 side effects were included to improve fit. The latent variable ‘virus’ relates to psychological perceptions of the COVID-19 virus, and ‘vaccine’ to the COVID-19 vaccine. Other abbreviations are outlined in Table 1 above.

The final model was an adequate fit for the data on some, but not all, indicators (CFI = 0.91; TLI = 0.87; SRMR = 0.09; RMSEA = 0.10). However, 49% of the variance in Booster Intention could be explained (*R^2^* = 0.49, *SE* = 0.03, *p* = <0.001). The full model is depicted in Figure 3. As was the case in the UK sample, both positive perceptions of the COVID-19 vaccine (*β* = 0.63, *SE* = 0.03, *p* < 0.001, 95% CI [0.57, 0.69]) and concern regarding the COVID-19 virus (*β* = 0.07, *SE* = 0.02, *p* = 0.005, 95% CI [0.02, 0.11]) positively predicted Booster Intention, while side effect severity to the second dose of the primary course (but not the first, see Model Stage 3, Table 4) negatively predicted Intention (*β* = −0.07, *SE* = 0.03, *p* = 0.018, 95% CI [−0.12, −0.01]). Several additional predictors were retained in the model. As the respondents age (*β* = 0.13, *SE* = 0.03, *p* < 0.001, 95% CI [0.08, 0.18]) and the number of months since their last COVID-19 vaccine increased (*β* = 0.08, *SE* = 0.02, *p* < 0.001, 95% CI [0.04, 0.12]), so did Intention. Conversely, self-report familiarity with the side effects of the three COVID-19 booster vaccines on offer decreased Intention (*β* = −0.05, *SE* = 0.02, *p* = 0.014, 95% CI [−0.09, −0.01]). Finally, those who reported receiving the flu vaccine yearly had higher Intentions than those who did not (*β* = 0.08, *SE* = 0.02, *p* < 0.001, 95% CI [0.04, 0.18]).

## 4. Discussion

The present study explored predictors of the intention to receive a COVID-19 booster vaccine. To achieve this, psychological perceptions of the COVID-19 virus and vaccine, alongside situational and contextual factors related to COVID-19, were measured in two nationally representative samples drawn from the UK and Australia. The prevalence of individuals who ‘definitely intended’ to receive a booster vaccine was high in both samples (73% and 67%, respectively). Similar high rates of acceptance have been reported regarding the primary vaccine course, estimated at 84% (UK) and 82% (Australia) in one recent meta-analysis [13] and 67% (Europe) and 76% (Oceana) in another [32], and are comparable to a recent global estimate (79%) for booster intent [17]. However, to the best of our knowledge, the present study is the first to measure booster intentions in Australia specifically [17]. Three primary variables emerged across both samples as significant predictors of COVID-19 booster intention: (1) concern regarding the COVID-19 virus; (2) perceptions of trust and efficacy regarding the COVID-19 vaccines; (3) the experience of side effects occurring to the most recent vaccine. Importantly, all three predictors have the potential to be targeted by behavioural intervention to encourage continued participation in the booster programme.

Consistent with the results of the present study, positive vaccine perceptions and concern regarding the virus have repeatedly been found to predict the intention to receive the first dose of a COVID-19 vaccination [23,33,34,35,36] as well as the booster vaccine [21,37,38]. These findings are reflected in systematic review and meta-analysis regarding the COVID-19 [17,32,39], influenza [40], and MMR [41] vaccination, demonstrating that psychological perceptions of this kind are key variables pertaining to vaccination intentions more broadly [12].

That trust and importance of COVID-19 vaccination predicted booster intention is consistent with evidence demonstrating that a lack of trust in the COVID-19 vaccines due to the speed of development and approval [42], or fears concerning adverse events and vaccine efficacy [35,43], increased initial COVID-19 vaccine hesitancy. As such, potential interventions that stress the safety, efficacy, and importance of COVID-19 vaccination may serve to maintain participation within a given COVID-19 vaccination programme. Evidence regarding the efficacy of such interventions is mixed; some studies have reported increased vaccine intent [44,45,46,47,48,49], and others not [50,51,52,53,54]. However, recent meta-analysis suggests that information regarding vaccine efficacy may be of particular relevance to those most hesitant [55]. Additional research provides preliminary evidence that emphasising vaccine efficacy may also increase COVID-19 vaccine uptake [56,57]. For any intervention of this type to be practical, it must be ensured that information is deployed in a transparent and ethical manner, without downplaying the risks of vaccination that may obscure patient informed consent. At the time of writing, only one study has addressed this issue, finding that transparency did not reduce the perception of vaccine efficacy, but failed to significantly modulate vaccine intent [58]. As such, it is unclear whether transparent communication diminishes any active benefit of this type of messaging on increased intention, with further controlled research employing matched comparators required. Additionally, as most interventions occurred during the early phases of vaccine development, a paucity of evidence exists regarding the interplay between interventions designed to increase vaccine perceptions and prior vaccine experience. Longitudinal research across all stages of the vaccine rollout (i.e., primary course and booster vaccination) is therefore needed.

Concern regarding infection with the COVID-19 virus, both personally and among close others, also increased booster intention in both UK and Australian samples. This is consistent with previous research concerning COVID-19 booster vaccine intent [17,59,60]. Interestingly, actual infection with COVID-19, both personally and among close others, did not predict intention in the UK sample (this was not analysed in the Australian sample). This replicates results reported elsewhere. Specifically, in these studies, worry or perceived threat associated with COVID-19 infection predicted booster vaccine intent, while actual infection did not [59,60]. This suggests that the affective dimension associated with potential infection may be particularly pertinent to booster intent. While many governments have sought to downplay the severity of COVID-19 [61,62], the virus continues to pose a risk to society [63], causing significant economic burden [64] and leaving individuals vulnerable to long-term health complications [65,66]. While instilling fear about COVID-19 would be a poor public health strategy, finding ways to combat COVID-19 complacency may play an important role in encouraging continued vaccination [62]. Several recommendations having been made in this domain, including the development of targeted and tailored messaging and interventions, delivered in multimodal presentation formats, to reduce pandemic-related fatigue [67,68].

Finally, the severity of side effects experienced to the most recent COVID-19 vaccine predicted a decreased intention to receive a booster vaccine in both samples. While one study reported limited association between side effects and booster intentions [21], several others have demonstrated a significant negative association [69,70,71]. One methodological difference is that the former enquired about specific instances of side effects, while the latter, like the present study, measured average side effect perceptions. Future research may therefore aim to disentangle whether these broader negative perceptions drive a decrease in vaccine intent, rather than specific memorable adverse events per se. If so, these perceptions may be easier to modulate given their generality across individuals. As most studies combined side effects across primary course doses [21,70,71], it is difficult to draw inference regarding the apparent recency effect observed in the present samples. However, such results appear not to be driven by an overall increase in the severity of side effects associated with the second dose as severity numerically decreased in both samples. Like all vaccines, vaccination against COVID-19 carries a risk of reactogenicity as a consequence of the body’s innate immune response [72]. However, recent evidence also implicates psychosocial processes in the experience of side effects, such as via the nocebo effect or misattribution of pre-existing symptoms. For example, up to 76% of systemic adverse events occurring in randomised controlled trials concerning COVID-19 vaccines could be attributed to the nocebo effect [73]. Negative expectations are believed to be a primary mechanism underpinning side effects of this type [74], with negative expectancies regarding COVID-19 vaccine side effects found to predict subsequent side effect experience [75]. Methods to reduce negative side effect expectations may therefore provide a route through which to encourage continued vaccination via a reduction in the perception of side effects occurring to previous doses. Current recommendations specific to COVID-19 involve providing accurate side effect information to build trust, employing positive framing when discussing adverse events, balancing risk and benefit information, and countering side effect-related misinformation [76].

An additional four predictors emerged from the Australian sample that were either not predictive (age, months elapsing since the last dose of a COVID-19 vaccine, perceived familiarity with vaccine side effects) or not measured (regular immunization with the flu vaccine) in the UK dataset. There are several reasons why these results may have been observed. For example, there were seasonal differences between samples, and different cumulative infection rates of COVID-19, with the UK experiencing a far greater number of infections at the time of data collection (although cumulative and daily increase in infection rates appear uncorrelated with COVID-19 vaccine acceptance in recent meta-analysis [16]). Clearly, both cross-sectional datasets are a snapshot of vaccine intention occurring within a specific context, with longitudinal research needed to reconcile potential differences in the predictors of vaccine intent across countries. However, we also note that the purpose of the present analysis was exploratory, with the intention of building parsimonious models that could be tested in future research. As such, we did not test for interactions between models, meaning that we cannot comment on whether the strength of predictors significantly differed between the two. We therefore review the evidence for the predictors found in the Australian model below, while remaining agnostic to any potential cross-cultural or temporal drivers of these effects. Specifically, examination of cross-cultural differences is clearly a pertinent question for future research, despite being complicated by the myriad of potential differences that could feed into models of this type. However, this was not the focus of the present study and did not feature in the pre-registered stats plan.

Consistent with results from the Australian sample, perceived familiarity with vaccine side effects have been found to decrease the intention to receive a primary course of a COVID-19 vaccine, with speculation that media discourse and misinformation may exacerbate the perceived risk of vaccination in this instance, lowering intention [33]. Consistent with this, we found that both samples overestimated the prevalence of COVID-19 vaccination side effects in our previously published research [18,19]. In both cases, we found that an intervention where positive attribute framing was applied to side effect prevalence increased booster intention [18,19] and reduced side effect worry and perceived severity [19]. The interaction between the attribute framing and perceived side effect familiarity was not analysed. Consequently, we cannot comment on whether this type of intervention is particularly effective among this subsample. However, given that attribute framing is ethical, cost effective, and easy to implement, it is likely to be an efficient method for reducing side effect concern. Regular immunisation with the influenza vaccine has previously been found to increase the intention to receive a primary COVID-19 vaccine course [34,35,77] and a booster vaccine [69,78]. This association is likely to bidirectional, with vaccination against COVID-19 also recently found to increase the intention to vaccinate against influenza [79]. Evidence regarding the effect of age (and other sociodemographic variables), however, has been variable with numerous studies finding limited association [22,33,37,38,80]. Finally, months elapsing since the last COVID-19 vaccine has not been tested in research investigating booster intent [17], although it follows that as immunity is perceived to wane, perceived risk should be elevated, causing an increased intention to be vaccinated.

There are several strengths to the present study, including the recruitment of nationally representative samples from two countries where limited evidence regarding booster vaccine intent presently exists [17]. While our results are broadly consistent with current evidence regarding COVID-19 vaccine intent, there are several limitations that should be noted. As stated, the cross-sectional data presented here provide a snapshot of vaccine intent measured during the initial onset of the ‘omicron’ wave and rollout of the booster vaccine programme in each country. Hesitancy regarding the COVID-19 vaccine is known to vary over time [81,82] and as such longitudinal research is required to track changes in the predictors of continued vaccination, including as new vaccines and changes in vaccine composition arise. Similarly, intention, but not uptake, was measured. Translation from intention to uptake is thought to be modulated by practical considerations, such as vaccine availability, cost, and access [83], with psychological determinants being the strongest predictors of intention and uptake where vaccines are accessible and affordable (i.e., as in the current samples) [12]. Empirically, vaccine intention has been demonstrated to be a strong predictor of vaccine uptake, e.g., [84,85,86], including for COVID-19 vaccination [87], although we do not assume that the two are synonymous [88]. However, at the time of publication, the intention to ‘definitely receive’ a COVID-19 booster vaccine in both samples (UK = 73%; Australia = 67%) tracked closely with the actual number of third doses administered in each country: ~75% and 69%, respectively (calculated as a percentage of the population over 16—the age at which boosters are available to those not classed as immunocompromised). While an averaged estimate, these number indicate that intentions may well map longitudinally with actual uptake. To confirm this, future research should not only measure intention and uptake but strive to uncover the factors at play should the two fail to cohere. Finally, the model conducted on the Australian dataset did not provide adequate fit on all metrics. We note that fit indices simply provide a rule of thumb and can lack reliability under estimation methods other than maximum likelihood [89]. However, that the predictors were consistent with previous research, could be replicated across models, and explained a significant proportion of the variance, provides some reassurance regarding the validity of the model.

In summary, the intention to receive a booster vaccine was found to be high in two nationally representative samples drawn from the UK and Australia. Three key variables emerged as predictors: psychological perceptions regarding the COVID-19 vaccine and virus as well as previously experienced side effects. A key insight is that all three variables have the potential to be modulated to encourage continued vaccination. The current research therefore makes a significant contribution to understanding the psychological and behavioural drivers of vaccine intent; a factor which continues to play an important role in the control and management of COVID-19.

## 5. Conclusions

In summary, while empirical evidence has amassed regarding the predictors of the intention to receive an initial COVID-19 vaccine, our knowledge of the factors associated with continued participation in the COVID-19 vaccination programme remains limited. Specifically, most published research has focused on sociodemographic predictors of COVID-19 booster vaccine intent, with few studies investigating psychological and contextual predictors related to the COVID-19 virus and vaccine. The current study addresses this critical gap in knowledge. The intention to receive a booster vaccine was found to be high in two nationally representative samples drawn from the UK and Australia. Three key variables emerged as predictors: psychological perceptions regarding the COVID-19 vaccine and virus as well as previously experienced side effects. A key insight is that all three variables have the potential to be modulated to encourage continued vaccination. The current research therefore makes a significant contribution to understanding the psychological and behavioural drivers of vaccine intent; a factor which continues to play an important role in the control and management of COVID-19.

## Figures and Tables

**Table 1:** Basic demographic information regarding the characteristics of the sample (additional information can be found in the following sources [18,19]) and descriptive statistics regarding the predictors in the UK and Australian models. These are presented as means (*M*) and standard deviations (*SD*), or frequency counts (*N*) and percentages (*%*), dependent on the type of measure (i.e., scale vs. categorical).

Demographic Information		
	UK Sample *N* (% of Sample)	Australian Sample *N* (% of Sample)
**Age bracket (years)**		
18–24	47 (3.8)	126 (10.5)
25–34	116 (9.5)	235 (19.6)
35–44	211 (17.3)	205 (17.1)
45–54	246 (20.1)	202 (16.9)
55–64	315 (25.8)	179 (15.0)
65+	287 (23.5)	250 (20.9)
**Gender**		
Woman	706 (57.8)	603 (50.4)
Man	511 (41.8)	592 (49.5)
Non-binary/other	5 (0.4)	2 (0.2)
**Employment**		
Employed full-time	407 (33.3)	494 (41.3)
Employed part-time	153 (12.5)	224 (18.7)
Self employed	98 (8.0)	45 (3.8)
Unemployed (looking)	38 (3.1)	62 (5.2)
Unemployed (not looking)/long-term sick or disabled	92 (7.5)	-
Parent/Carer	70 (5.7)	68 (5.7)
Student	24 (2.0)	43 (3.6)
Retired	321 (26.3)	256 (21.4)
Other	19 (1.6)	5 (0.4)
**Predictor Variables**		
	***M*** (**SD**)/***N*** (**%**)	***M*** (**SD**)/***N*** (**%**)
**BeSD1** (*How concerned are you about getting COVID-19?*)	50.5 (31.3)	47.7 (30.4)
**BeSD2** (*How concerned are you about your close family and friends getting COVID-19?*)	60.6 (31.0)	56.3 (31.3)
**BeSD3** (*How much do you trust the COVID-19 vaccines?*)	80.0 (22.6)	73.9 (24.6)
**BeSD4** (*How important do you think getting a COVID-19 vaccine will be for your health?*)	77.5 (24.6)	75.7 (24.7)
**BeSD5** (*How much do you think getting a COVID-19 vaccine for yourself will protect other people in your community?*)	86.1 (22.4)	81.1 (23.0)
**Months Vax** (*Months since last COVID-19 vaccine*)	4.8 (1.5)	3.4 (2.3)
**Dose1SE** (*Previous vaccine side effects—dose 1*)	21.3 (27.9)	23.3 (25.4)
**Dose2SE** (*Previous vaccine side effects—dose 2*)	13.7 (21.7)	21.7 (25.1)
**Familiarity** (*familiarity with COVID-19 vaccine side effects*)	48.2 (28.3)	52.3 (22.8)
**Age**	52.5 (14.5)	47.3 (17.2)
**Vax status** (*primary course of vaccination: 0 = AstraZeneca/1 = Pfizer*)	*AZ* = 615 (50.3%)	*AZ* = 503 (42.0%)
**COVID Self** (*personal infection with COVID-19: 0 = no/1 = yes*)	*yes* = 148 (12.1%)	-
**COVID Other** (*infection among close others: 0 = no/1 = yes*)	*yes* = 565 (46.2%)	-
**Flu Vax** (*previous experience with the flu vaccine: 1 = yearly/0 = other*)	-	*yearly* = 545 (45.5%)
**Immunocompromised** (*Are you regarded as immunocompromised by your GP: 0 = no/1 = yes*)	-	*yes* = 117 (9.8%)

**Table 2:** Model fit for the initial EFA performed on the selected BeSD items.

	UK Sample		Australian Sample	
EFA: Model Fit				
	One-Factor	Two-Factor	One-Factor	Two-Factor
CFI	0.57	1.0	0.50	1.0
TLI	0.14	0.99	0.01	1.0
SRMR	0.18	0.003	0.19	0.001
RMSEA	0.38	0.03	0.40	<0.001

**Table 3:** Predictors pruned from the model at each stage of model refinement (UK sample).

Predictive Value of the Variable Pruned from the Model				
**Variable**	***β***	**S.E.**	***p***	**95% CI**
Model Stage 1: Age	−0.01	0.03	0.78	[−0.07, 0.06]
Model Stage 2: COVID other ^1^	−0.01	0.02	0.70	[−0.05, 0.03]
Model Stage 3: COVID self ^2^	−0.01	0.02	0.64	[−0.06, 0.04]
Model Stage 4: Familiarity ^3^	−0.10	0.02	0.61	[−0.05, 0.03]
Model Stage 5: Dose1SE ^4^	0.03	0.03	0.43	[−0.04, 0.09]
Model Stage 6: Months vax ^5^	0.03	0.03	0.19	[−0.02, 0.09]
**Improvement in Model Fit after Variable Removal**				
**Model Comparison**	**CD ^6^**	**TRd ^7^**	**Δdf**	***p* (for TRd/Δdf)**
Full Model vs. Stage 1	1.10	57.13	5	<0.001
Stage 1 vs. Stage 2	0.99	9.74	5	0.083
Stage 2 vs. Stage 3	1.25	26.91	5	<0.001
Stage 3 vs. Stage 4	0.98	35.75	5	<0.001
Stage 4 vs. Stage 5	1.22	23.74	5	<0.001
Stage 5 vs. Stage 6	1.07	60.72	5	<0.001

^1^ Experience of close others being infected with the COVID-19 virus (0 = no experience/1 = experience), ^2^ Personal experience of being infected with the COVID-19 virus (0 = not experienced/1 = experienced), ^3^ Average familiarity with the side effects of the COVID-19 vaccines, ^4^ Side effects experienced to the first dose of the vaccine primary course, ^5^ Months since last COVID-19 vaccine, ^6^ Difference Test Scaling Correction (CD) associated with the Satorra-Bentler method, ^7^ Satorra-Bentler Scaled Chi-Square Difference (TRd).

**Table 4:** Predictors pruned from the model at each stage of model refinement (Australian sample).

Predictive Value of the Variable Pruned from the Model				
**Variable**	***β***	**S.E.**	***p***	**95% CI**
Model Stage 1: Vax Status ^1^	−0.004	0.03	0.89	[−0.06, 0.06]
Model Stage 2: Immunocompromised ^2^	−0.02	0.02	0.30	[−0.06, 0.02]
Model Stage 3: Dose1SE ^3^	0.03	0.03	0.22	[−0.02, 0.08]
**Improvement in model fit after variable removal**				
**Model Comparison**	**CD ^4^**	**TRd ^5^**	**Δdf**	***p* (for TRd/Δdf)**
Full Model vs. Stage 1	1.06	24.10	5	<0.001
Stage 1 vs. Stage 2	1.00	20.55	5	0.001
Stage 2 vs. Stage 3	1.09	37.31	5	<0.001

^1^ Primary course of the AstraZeneca or Pfizer vaccine, ^2^ Status as immunocompromised (1 = immunocompromised/0 = not), ^3^ Side effects experienced to the first dose of the vaccine primary course, ^4^ Difference Test Scaling Correction (CD) associated with the Satorra-Bentler method, ^5^ Sattora-Bentler Scaled Chi-Square Difference (TRd).

## Data Availability

Data is available from the following source: https://osf.io/xbkd2/ (accessed on 2 September 2022).
